# Primary Cilium in Cancer Hallmarks

**DOI:** 10.3390/ijms20061336

**Published:** 2019-03-16

**Authors:** Lucilla Fabbri, Frédéric Bost, Nathalie M. Mazure

**Affiliations:** Université Côte d’Azur (UCA), INSERM U1065, C3M, 151 Route de St Antoine de Ginestière, BP2 3194, 06204 Nice, France; lucilla.fabbri@unice.fr (L.F.); bost@unice.fr (F.B.)

**Keywords:** autophagy, cancer hallmarks, cell cycle, hypoxia, primary cilium, signaling pathways

## Abstract

The primary cilium is a solitary, nonmotile and transitory appendage that is present in virtually all mammalian cells. Our knowledge of its ultrastructure and function is the result of more than fifty years of research that has dramatically changed our perspectives on the primary cilium. The mutual regulation between ciliogenesis and the cell cycle is now well-recognized, as well as the function of the primary cilium as a cellular “antenna” for perceiving external stimuli, such as light, odorants, and fluids. By displaying receptors and signaling molecules, the primary cilium is also a key coordinator of signaling pathways that converts extracellular cues into cellular responses. Given its critical tasks, any defects in primary cilium formation or function lead to a wide spectrum of diseases collectively called “ciliopathies”. An emerging role of primary cilium is in the regulation of cancer development. In this review, we seek to describe the current knowledge about the influence of the primary cilium in cancer progression, with a focus on some of the events that cancers need to face to sustain survival and growth in hypoxic microenvironment: the cancer hallmarks.

## 1. Introduction

The primary cilium is a solitary organelle that consists of a microtubule core, the axoneme, and extends from the cell surface into the extracellular environment growing from the basal body, a structure that is derived from centrioles. Although the structure and functions of the primary cilium are now well-established (even if they are under continuous investigation), our current knowledge about primary cilia is founded from a long course of research, starting from the end of the 19th century. At that time, the first reports of primary cilia came from studies on sea chordates, in which the primary cilia were found to occur singly on cells of different epithelia [1]. Among researchers, it is worth remembering Paul Langerhans [2], who was best known for his discoveries of the Langerhans cells of the skin and the Islets of Langerhans of the pancreas. The most famous scientist who is usually credited for the discovery of primary cilia was Karl Wilhelm Zimmermann. Even though he was not the first to observe primary cilia, he receives the credit for the discovery of primary cilia in mammalian cells (including humans) [3]. Moreover, in his paper dated from 1898, he noted that primary cilia on rabbit kidney tubule epithelial cells were always associated with a pair of centrioles, and in particular, with the closest centriole to the plasma membrane. He was also the first to speculate about the sensory functions of primary cilia in kidney epithelial cells. Still in 1898, the heavily debated Henneguy–Lenhossek hypothesis argued that centrioles of the centrosome and basal bodies of ciliated cells shared the same identity [4,5]. The work of Cowdry is also of note, where, in 1921, he described the absence of motility for the “flagella” of the luminal surface of thyroid follicle cells of the dogfish, but precisely because they were nonmotile, no functions were ascribed to them [6]. Until the 1950s, primary cilia had somehow been “forgotten”, but after this period, studies on primary cilia returned to the forefront.

In between the 1950s and 1960s, the development of electron microscopy (EM) brought the occurrence of primary cilia into the light, across a variety of tissues in which primary cilia had never before been seen (neurons and lung), as well as the internal 9 + 0 arrangement of the microtubules within the axoneme, which were continuous with the microtubules of the distal centriole of the pairs [7,8,9,10,11,12]. Studies on ciliary structure began with Keith R. Porter, one of the pioneers of EM, who initially characterized the internal 9 + 2 organization of motile cilia in a comparative study on mollusks, amphibians, and mammals [13]. In 1956, deHarven and Bernhard demonstrated the correspondence between basal bodies and centrioles [14]. Twelve years later, Sorokin proposed the “primary cilia” term that we currently use to define the appendage that was first seen to emerge from the epithelial cells of the developing mammalian lungs, before the formation of the motile cilia that make up the ciliated border. He also carried out a huge study on the comparison between the ciliogenesis of primary and motile cilia [15,16]. Given the similarity between the 9 + 0 structure of primary cilia, and the sensory structures of the photoreceptor-connecting cilium [17], a sensory function for primary cilia was only supposed at that time [8].

The first demonstration of the mechanosensory function of fluid flow for primary cilia in kidney cells was shown in 2001 [18], a century after Zimmermann’s first proposition in this sense. The discovery of the intraflagellar transport (IFT) in the flagella of the green alga *Chlamydomonas* was revolutionary [19], demonstrating bidirectional movement of particles along ciliary and flagellar microtubules, and its further involvement in cilia assembly and disassembly [20].

It was therefore easy to speculate that defects in the structure of these organelles could lead to important diseases. In 2000, Pazour gave the first demonstration that primary cilia were involved in many human disorders, in a mouse model for autosomal dominant polycystic kidney disease (ADPKD) [21,22]. His work paved the way for copious studies linking many different diseases that affect all body tissues (i.e., obesity, mental retardation, retinal defects and cancer) to primary cilia defects: the so called “ciliopathies” (reviewed in [23,24]).

Nowadays, thanks to this fundamental literature, we can appreciate the many facets of the primary cilium that we are still discovering, as well as its fundamental importance in all human organs. Its functions spread from the perception of light and odorants to mechanosensation, and importantly, coordination and the transduction of a number of signaling pathways (reviewed in [25]). So far, a wide spectrum of ciliary proteins constituting the cilium proteasome have been characterized [26], and among these, some proteins that function in modulating the transduction of cancer-linked molecular signals, such as Smoothened (SMO) [27], Platelet-Derived Growth Factor Receptor (PDGFR) [28] and Vang-like protein 2 (VANGL2) [29] among others, which have been given much attention regarding the role of primary cilia in cancer. Given the function of the primary cilium as a control center for signaling pathways associated with tumorigenesis, such as Hedgehog (HH), Wnt, and PDGF signaling pathways, as well as its close relationship with the cell cycle [30], both the presence or loss of the primary cilium by the cells can be crucial in a tumor context.

In this review, we attempt to describe what it is currently known about the involvement of primary cilia in cancer, focusing mostly on the well-established cancer hallmarks [31], which are essential elements for cancer outgrowth and survival.

## 2. Ciliogenesis as a Timeout for Cell Cycle Progression

Uncontrolled cell proliferation and deregulation of the cell cycle are hallmarks of cancer cells and neoplastic development. In this section, we describe how the genesis of the primary cilium is closely related to the cell cycle, and how it can control its progression.

### 2.1. Primary Cilia and the Cell Cycle

**Figure 1:**
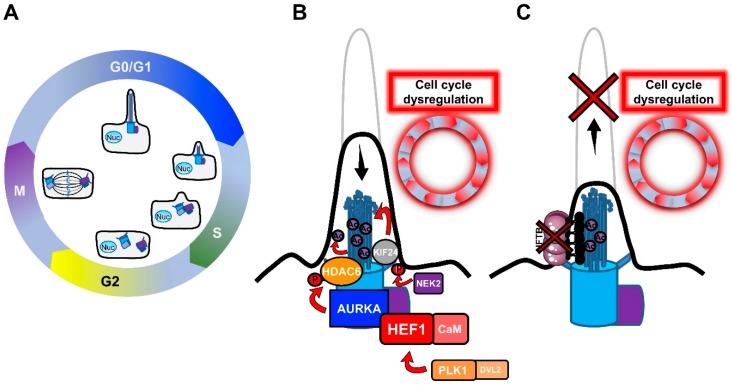
Regulation of ciliogenesis and cell cycle. (**A**) Primary cilium formation occurs during the G0/G1 phase. Upon entry into S phase, the DNA, and the mother and daughter centrioles (blue and purple boxes respectively) initiate replication, and two newly centrioles are formed. Before mitosis, the new pair of centrioles migrate to the opposite pole of the cell, and the daughter centriole matures into a new mother centriole. Ciliary disassembly takes place at the G2/M transition. After mitosis, each daughter cell inherits a pair of centrioles, and the cilia reassemble in the next G0/G1 phase. (**B**) Cell cycle regulators AURKA, PLK1, and NEK2 participate in cilium disassembly, thus impairing the cell cycle. This may explain the involvement of these factors in cancer progression. HEF1/CaM binds to AURKA, promoting its activation. AURKA in turn phosphorylates and activates HDAC6, resulting in HDAC6 mediated deacetylation of substrates in the ciliary axoneme, causing ciliary resorption. PLK1/DVL2 can also activate HEF1, and NEK2 phosphorylates KIF24, which promotes microtubule disassembly. (**C**) The disruption of the anterograde IFTB subcomplex and/or the kinesin molecular motor leads to defects in the cilium assembly, thus promoting cell cycle defects and cell proliferation. Ac: Acetylation; AURKA: Aurora A Kinase; CaM: Calmodulin; DVL2: Dishevelled segment polarity protein 2; HDAC6: Histone deacetylase 6; HEF1: Human enhancer of filamentation 1; IFTB: Intraflagellar transport B; KIF24: Kinesin family member 24; NEK2: Never in mitosis A (NimA) related kinase; Nuc: Nucleus; P: Phosphorylation; PLK1: Polo-like kinase 1.

The relationship between primary cilia and the cell cycle was recognized early in the long history of primary cilia, with the observation of primary cilium resorption before mitosis [15,16,30,32,33]. In most mammalian cells, the primary cilium is assembled in the post-mitotic G0/G1 phases of the cell cycle, and disassembled before mitosis, in intimate association with the centriole cycle (Figure 1A).

The reason for this dependence relies on the fact that the ciliary microtubule axoneme extends from the cell surface by anchoring to the basal body. The basal body is derived from the oldest of the two centrioles of the centrosome, the mother centriole, as a result of a process of maturation consisting of the acquisition of distal and sub-distal appendages [34,35,36]. The centrosome/basal body has a barrel-shaped structure made up of nine triplet microtubules that, during ciliogenesis, act as a template for the axonemal growth [37]. Since the centrosome serves as a microtubule-organizing center (MTOC) for the generation of the mitotic spindle during mitosis [38,39,40], a common thought is that ciliogenesis only initiates when the centrosome is free from its functions during cell division. As shown in Figure 1A, the mother and the daughter centrioles of the centrosome undergo replication during S phase in dividing cells, resulting in the formation of two new centrioles that remain associated with the pre-existing ones. Before mitosis, the mother and the daughter centrioles migrate to the opposite poles of the cell, together with the newly-formed centrioles, to set up the mitotic spindle. At this point, the daughter centriole acquires additional appendages to become a new mother centriole. After cell division, each daughter cell asymmetrically inherits a pair of centrioles, one containing the old mother centriole and one containing the new mother centriole [41]. It has been shown that the asymmetrical inheritance of centrioles influences the timing of cilium formation in the new daughter cells once they enter in the next G0/G1 phase [42]. According to the double function of the centrosome (as the MTOC, during mitosis, and basal body, the template for ciliogenesis), defects in cilium formation can deregulate the cell cycle, or inversely, the persistence of the primary cilium can put a brake on it, forcing the cells to enter a quiescent state. In favor of this statement, many cancer cells, which are characterized by a high proliferation rate, lack a primary cilium [43,44,45,46,47,48], but whether this lack is the cause or the consequence of transformation is not well understood. The interchange between ciliogenesis and cell division is accentuated by the fact that many cell cycle regulators play a critical role in cilium fate and, among these, Polo-like kinase 1 (PLK1), Aurora A Kinase (AURKA) and Never in mitosis A (NimA)-related kinase (NEK2) were found to be implicated in tumorogenesis (Figure 1B).

- PLK1 is a mitotic kinase that regulates progression through the cell cycle by phosphorylating serine/threonine proteins on centrosomes, kinetochores, the mitotic spindle, and the midbody [49]. Activation of the non-canonical Wnt pathway induces the formation of the Plk1- disheveled segment polarity protein 2 (Dvl2) complex, which activates AurkA through the stabilization of Human Enhancer of Filamentation 1 (HEF1), thus inducing cilium disassembly [50].
- AURKA is a centrosomal mitotic kinase that regulates S phase entry. After mitosis, it localizes to the basal body, and is activated by the scaffold protein HEF1 and calmodulin (CaM) in the presence of calcium [51]. The HEF1–Ca^2+^/CaM–AURKA complex in turn activates the tubulin deacetylase histone deacetylase 6 (HDAC6), which destabilizes axonemal microtubules, inducing cilium disassembly [52].
  AURKA is the point of convergence between these two pathways, and cilium disassembly occurs downstream of its activation. AURKA was found to be upregulated in non-ciliated ovarian and clear cell renal cell carcinoma cancer cells [44,45], and HDAC6 inhibition restored primary cilia in chondrosarcoma and cholangiocarcinoma cancer cells, suppressing cell proliferation and their invasion capacity [53,54]. Similarly, HEF1 overexpression was associated with the metastasis of breast cancer and melanoma [55,56].
- NEK2 is another important regulator of both centrosome and basal body [57]. NEK2 exerts its role in the disassembly of the axonemal microtubules by phosphorylating the Kinesin Family Member 24 (KIF24), a member of the kinesin superfamily of microtubule-based motor proteins, which stimulates its microtubule-depolymerizing activity and prevents the formation of cilia in proliferating cells [58]. NEK2 and KIF24 were found to be overexpressed in breast cancer cells, and ablation of these proteins restored ciliation, thereby reducing proliferation.

Thus, the formation of the primary cilium seems to be strictly regulated by the cell cycle, in which the centrosome acts as a key checkpoint. On the other hand, the primary cilium is important for proper cell cycle progression, by restricting it, which may have important implications for cancer development.

### 2.2. Intraflagellar Transport (IFT)

Axonemal outgrowth, turnover, and disassembly are mediated by the IFT multisubunit complexes, which were first discovered in *Chlamydomonas* [19]. In the green alga, IFT was identified by differential interference-contrast microscopy as granule-like particles that moved along the flagella microtubules, from the base to the tip (anterograde transport), and back from the tip to the base (retrograde transport). Later studies on *Chlamydomonas* biochemically characterized two distinct subcomplexes, IFTA and IFTB, as well as their constituent proteins [59,60]. The IFT-A subcomplex is made up of six proteins (IFT144, 140, 139, 122, 121, and 43), whereas 16 subunits are found in IFT-B (IFT172, 88, 81, 80, 74, 70, 57, 56, 54, 52, 46, 38, 27, 25, 22, and 20). Mutagenesis experiments in green alga have been able to identify kinesin-2 and dynein 1b as the molecular motors catalyzing, respectively, the anterograde and retrograde transport of IFT particles, with IFTB contributing to the anterograde, and IFTA to the retrograde transport [61,62,63,64,65,66]. A detailed study using a combination of correlative total internal reflection fluorescence (TIRF) and three-dimensional (3D) EM clarified how IFT trains avoid collisions during the bidirectional trafficking [67]. Stepanek and Pigino analyzed IFT trains with a high spatiotemporal resolution, showing that, despite sharing the same microtubule doublet, anterograde trains were associated with the B-microtubule and retrograde trains were associated with the A-microtubule of the doublet, thus avoiding crashes. The same group has recently found that the transition between anterograde and retrograde transport is elegantly coordinated by the IFT trains themselves. They showed that the retrograde motor dynein-1b is loaded onto IFTB in an auto-inhibited form, and in a precise spatial configuration that hampers its binding to the microtubule track, there by preventing its motor activity until it reaches the ciliary tip. Once at the ciliary tip, dynein-1b must be activated to initiate retrograde transport, but the mechanism of dynein-1b activation at the ciliary tip is still not known [68]. Early studies in *Chlamydomonas* demonstrated that new axonemal subunits are added at the distal end of the growing axoneme [69], and that this task is accomplished by the anterograde IFTB, which transports tubulin (α and β heterodimers) from the site of synthesis in the cytoplasm to the ciliary tip. Among the multiple particles composing IFTB, IFT81 was found to bind the globular part of the tubulin dimers through its calponin-homology (CH) domain, whereas its binding partner, IFT74, increases the affinity for tubulin through electrostatic contacts [70]. Since at least two tubulin-binding sites within the IFT complex were thought to be required to sustain the fast initial flagellar assembly rate, another tubulin/microtubule binding site was identified as the CH domain of IFT54 [71]. Craft et al. observed that IFT particles were overloaded with tubulin during axonemal growth, in order to concentrate tubulin within the ciliary matrix, and to facilitate the axonemal polymerization [72].

It has been shown that the knock-down of the IFT27 protein affected both flagellar assembly and the cell cycle, causing defects in cytokinesis [73]. Similarly, the depletion of IFT20 unblocked G0/G1 arrest, through cilia disruption [74]. With the work of Pazour et al. came the first observation that disruption of ciliary formation by targeting IFT results in the development of polycystic kidney disease (PKD), a disorder that is characterized by the onset of kidney cysts with increased proliferation [22]. Additional evidence for IFT-induced ciliary assembly defects in PKD was provided by an analysis of targeted knockouts of the KIF3A subunit of kinesin2 anterograde motor in kidney epithelium [75]. Thus, IFT, by functioning in building primary cilia, may contribute to slowing down the cell cycle by arresting cell proliferation.

All these findings suggest that IFT is one of the limiting factors, together with cellular tubulin availability, for cilium formation, and that IFT can thus influence cell cycle progression (Figure 1C). Besides its function in assembling cilia, IFT also plays a direct role in cilium-mediated cell signaling, which can directly influence tumorigenesis. The role of IFT/primary cilium and cell signaling in cancer will be discussed in detail in the next paragraph (§3).

## 3. Primary Cilium as a Mediator of Signaling Pathways

Signaling pathways that are involved in normal cellular growth and tissue development are deregulated in all stages of oncogenesis. In tumors, altered signaling pathways mediate resistance to cancer therapy, cell death, and evasion to immunosurveillance. The primary cilium, by sensing signals from the extracellular environment, and by displaying both the protein receptors required for signal interception, as well as the downstream molecular effectors, is a key mediator of the impaired signaling that induces malignancy. Among the pathways associated with the primary cilium, the Hedgehog, Wnt, and PDGF signaling pathways are well-characterized.

### 3.1. Hedgehog Signaling Pathway

**Figure 2:**
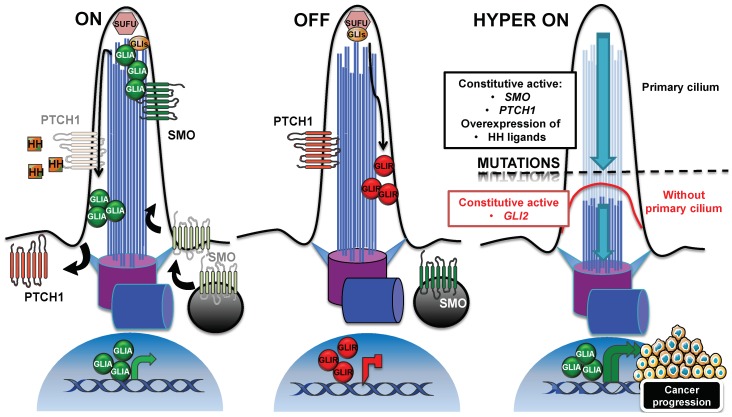
Cilia-dependent regulation of the Hedgehog signaling pathway. Schematic representation of the Hedgehog pathway repression and activation, in the presence or absence of a ligand. In Panel “ON”, in the presence of ligand binding to PTCH1 receptor, SMO moves into the cilium, and PTCH1 is displaced outside. In the cilium, SMO inhibits SUFU, and it activates GLI transcription factors (GLIA), which, in turn, activate the expression of target genes. In contrast, in Panel “OFF”, in the absence of a ligand, PTCH1 receptor localizes at the primary cilium, and the 7-transmembrane domain protein SMO is in vesicles outside the cilium. GLI transcription factors are sequestered and suppressed by SUFU, and processed into their repressive forms (GLIR), thus repressing the expression of target genes. If the pathway is misregulated, “HYPER ON” (i.e., *SMO*, *PTCH1* mutations, in medulloblastoma and basal cell carcinoma), the activation of the pathway throughout the cilium can induce tumorigenesis, and targeting of the cilium can turn off the pathway and reduce cell proliferation. On the other hand, if the misregulation of the pathway arises downstream (i.e., *GLI2* mutations), the loss of the cilium can also boost medulloblastoma or basal cell carcinoma development, suggesting that the role of primary cilia in Hedgehog misregulation-driven cancers depends on the oncogenic alteration. GLI: GLI family zinc fingers; PTCH1: Patched-1; SMO: Smoothened; SUFU: Suppressor of Fused.

Through the primary cilium, the Hedgehog signaling pathway is activated by ligands that are secreted by cells in tumors, which respond in an autocrine–juxtacrine manner [76]. Alternatively, ligands secreted by tumor cells can activate the signal into the surrounding tumor microenvironment in a paracrine manner, or vice versa [77,78]. In mammals, the three members of Hedgehog (HH) family are Sonic HH (SHH), Indian HH (IHH), and Desert HH (DHH), of which SHH is the best-characterized. Briefly, as shown on Figure 2 (panel “ON”), the cilium-mediated pathway is activated through the binding of HH to its receptor PTCH1 on the ciliary membrane [79]. In the cilium, PTCH1 prevents the ciliary localization of the seven transmembrane protein Smoothened (SMO), which localizes outside the cilium in vesicles close to the cell membrane [27]. After ligand-binding to PTCH1, SMO accumulates in the cilium, and PTCH1 is displaced outside [80], although its removal from the cilium is not critical for pathway activation [79]. In the cilium, SMO acts by activating the GLI family of zinc-finger transcription factors, GLI1, GLI2, and GLI3, which are effectors of the pathway. In absence of the ligand, GLI proteins are processed into repressive forms, in order to shut down the pathway [81,82] (Figure 2, panel “OFF”). Suppressor of fused (SUFU) is a negative regulator that prevents the aberrant activation of GLIs. Both the inactive GLIs and SUFU localize to the ciliary tip [83]. When activated, GLI proteins trigger the transcription of genes that are involved in proliferation, survival and epithelial-to-mesenchymal transition (N-Myc, Cyclin D/E, Bcl2, Snail, Slug, and FOXC2 are some examples), and are thus essential for tumorigenesis and cancer progression [84].

The requirement of IFT in the Hedgehog pathway is well-established, and most studies were carried out in the context of mice embryogenesis. *Wimple*, *flexo*, and *Kif3a* mouse mutants showed abnormal embryonic morphologies at the neural tube level, which recalled the phenotype induced by deficient hedgehog signaling. *Wimple* and *flexo* mutations corresponded to genes homologous to *Chlamydomonas IFT88* and *IFT172*, two components of the IFTB subcomplex. All mutants showed a reduction in hedgehog signaling, and all three proteins were identified to be required in the pathway at a step downstream to Ptch1, but upstream to Glis [85]. A similar aberrant phenotype during embryonic development was caused by a partial loss of function of Ift52 [86], or by mutations in two subunits of the retrograde dynein IFT motor [87]. Further experiments on mice showed that Ift proteins are a requisite for proper Gli activation and repression, in response to Hedgehog ligands [88].

All these findings indicate that the primary cilium and IFT are indispensable for the Hedgehog signaling pathway function, but whether IFT directly participates to the transport of Hedgehog components, or whether the loss of Hedgehog responsiveness is an indirect effect of IFT mutant-induced impairment of ciliogenesis still remains open to question. Recent findings based on a rapamycin-induced in-cell dimerization system to sequester IFT proteins into mitochondria showed that disrupted IFT did not affect SMO accumulation in the cilium, suggesting that SMO enters the cilium in an IFT-independent manner [89]. In effect, it has been shown that SMO preferentially moves to the cilium by diffusion within the membrane [90].

Another essential protein was found to mediate the trafficking of Hedgehog components within the cilium: the ADP ribosylation factor-like GTPase 13B (ARL13B), which is extensively used as a primary cilium marker [91]. Mutant *Arl13b^hnn^* MEFs failed to increase the Hedgehog response after Shh stimulation, and displayed an altered distribution of Hedgehog components, such as Gli2, Gli3, Sufu, and also Smo, which was found to be enriched in the cilia, even in the absence of Hedgehog stimulation. Thus, in contrast to that reported above, SMO entry into the cilium seems to be mediated by ARL13B, which is also essential for the proper distribution of SMO within the cilium after pathway activation. Inactivating mutations in *PTCH*, *SUFU*, and *GNAS* or activating mutations of *SMO*, indicative of Hedgehog pathway misregulation, have been described for sporadic basal cell carcinoma and medulloblastoma tumors [92,93,94,95].

On the basis of these findings, which support a role for the cilium in correct pathway activation, we may assert that, for the Hedgehog-driven tumor, the presence of the primary cilium can boost tumorigenesis and cancer progression (Figure 2, panel “HYPER ON”), contrary to the example in the previous section. Bay et al. demonstrated in a recent study that loss of Arl13b abolished cilia-mediated Hedgehog signaling in vitro, but also in vivo in Ptch-deficient mice, where the development of medulloblastoma was arrested [96].

Other studies on mice, however, showed that in the context of Hedgehog pathway, the primary cilium can either boost or inhibit tumorigenesis. Wong et al. assessed the role of primary cilia in mouse basal cell carcinoma [97] and found that the expression of a conditional allele of a constitutive active form of Smo induced a highly proliferative and ciliated basal cell carcinoma, in which the constitutive active Smo was localized to the cilia. In contrast, cilia were resorbed, following the expression of the conditional *kif3a* loss-of-function allele and cilia loss turned the Hedgehog pathway “off”, by decreasing cell proliferation. However, the expression of a constitutively-active human GLI2, in concomitance with the loss of Kif3a-induced cilia resorption, did not protect against tumor formation, but instead drove faster neoplastic growth, with a hyperactivation of Hedgehog signaling (Figure 2, panel “HYPER ON”). Moreover, the loss of cilia in basal cell carcinomas, initiated by constitutively active Gli2, was responsible for the disruption of the repressive form of Gli3 that was associated with an upregulation of Gli effectors. Han et al. came to a similar conclusion in a study focused on mouse medulloblastoma [98]. They showed that the cilia in the granule neuron precursors induced tumorogenesis in Smo-driven medulloblastoma, whereas cilia loss was required for medulloblastoma growth by constitutively active Gli2. Accordingly, the partial loss of cilia in heterozygous *ift88^+/−^* mice overexpressing Gli2 induced an increase in Hedgehog signaling and cartilage tumor incidence [99], and the inhibition of ciliogenesis increased the expression of Hedgehog target genes, and the metastatic potential of a mouse breast cancer, attributed to the inability to process the repressor form of the Gli transcription factor [100]. In line with the importance of primary cilia in the generation of the GLI repressor forms, which are necessary for the shut-down of Hedgehog, was the discovery of the cilium-dependent function of the conserved vertebrate G-protein-coupled receptor (GPCR) Gpr161 [101]. The ciliary Gpr161 mediated the processing of Gli3 in a cAMP-dependent manner, by acting as an Hedgehog pathway antagonist. *Gpr161* knockout mouse embryos displayed an increase in Hedgehog target genes that were aberrantly localized in the developing neural tube, concomitant with a decrease in the expression of the Gli3 repressor form. In addition, Shimada et al. demonstrated a tumor-suppressive role, played by Gpr161 in the context of a Shh medulloblastoma subtype [102]. The conditional depletion of Grp161 in the granule neuron precursors or in the neural stem cells of the mouse embryos induced an increase in the expression of Hedgehog target genes, while reducing the Gli3 processing in its repressor form. The increase of Hedgehog signaling gave rise to a cilium-dependent overproliferative phenotype of granule neuron precursors, and finally to tumorigenesis.

These findings indicate that the primary cilium, by directing the Hedgehog signaling pathway, can act both as a promoter of tumorigenesis, or as a tumor suppressor, depending on the initiating oncogenic alteration, thereby influencing cancer development in opposing ways.

### 3.2. PDGFRα Signaling Pathway

**Figure 3:**
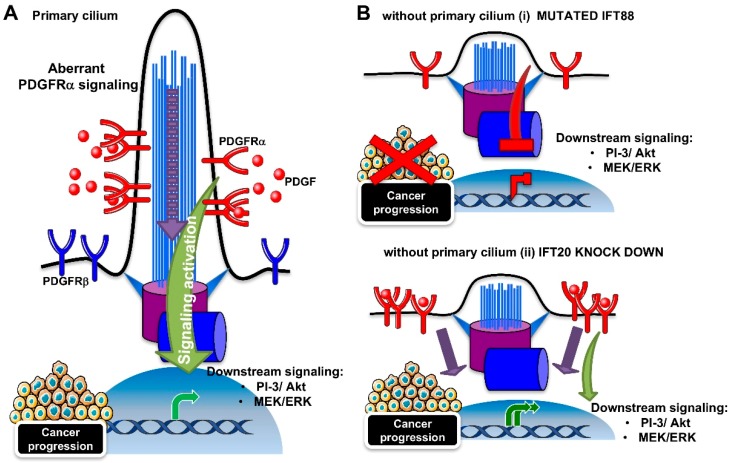
Cilia-dependent regulation of PDGFRα signaling pathway. (**A**) Schematic representation of the cilia-dependent PDGFRα signaling pathway. The α isoform of the PDGFR receptor is located at the ciliary membrane, whereas the β isoform is on the plasma membrane. The binding of the PDGF ligand to the PDGFRα receptor activates downstream signaling, such as PI-3/Akt and MEK/ERK. The aberrant activation of the pathway induces tumorigenesis. (**B**) The absence of the cilium can (i) repress the aberrant PDGFRα signaling pathway, thus potentially inhibiting tumorigenesis, or (ii) hyperactivate the pathway, due to a PDGFRα mislocalization to the plasma membrane, thus expanding the signal and inducing tumorigenesis. ERK: Extracellular signal–regulated kinase; IFT: Intraflagellar transport; PDGF: Platelet-derived growth factor; PI-3: phosphoinositide-3; PDGFRα Platelet-derived growth factor receptor α.

The PDGFRα signaling is the best described pathway among the receptor tyrosine kinase pathways, for its coordination by the primary cilium. The pathway depends on two receptors (PDGFRα and PDGFRα) and four ligands (PDGF A-D). As shown in Figure 3A, the binding of the ligand induces homo- or hetero-dimerization, and subsequent autophosphorylation on the tyrosine residues of the receptors. The phospho-tyrosines act as platforms for the binding of downstream molecules that trigger downstream signaling, such as mitogen-activated protein kinases (MAPK) and PI-3/Akt kinase pathways [103]. In growth-arrested NIH3T3 fibroblasts, PDGFRα localizes to the primary cilia [28]. The β isoform of the receptor was contrarily found along the cell surface. Serum starvation and ligand stimulation lead to an increase in PDGFRα protein expression and activation, as measured by tyrosine phosphorylation and the activation of Akt and Mek1/2-Erk1/2 pathways, in correspondence with cilium elongation. Ciliary localization was also found for the components of the downstream signaling cascade (Mek1/2). However, cilia-lacking fibroblasts derived from *Tg737^orpk^* mice with mutated *Ift88* were not able to increase PDGFRα levels and activity, neither in the case of serum-induced growth arrest, nor ligand stimulation (Figure 3B (i)).

Since aberrant PDGFRα signaling is linked to many human tumors, such as gliomas, osteosarcoma, and gastrointestinal stromal tumors [103], it can be argued that the suppression of the primary cilium can be beneficial for cancer regression. However, a recent study showed that the downregulation of IFT20, with a consequent loss of cilia, does not suppress PDGFRα signaling, but in contrast, it extends it [104] (Figure 3B (ii)). At the cilium, the IFT20 protein was found to interact with the E3 ubiquitin ligases, c-Cbl, and Cbl-b, where they participate in the ubiquitination and internalization of the PDGFRα receptor, in order to switch off the pathway. However, IFT20 depletion caused the autoubiquitination and degradation of c-Cbl, with a mislocalizaton of PDGFRα to the plasma membrane, thus impairing negative feedback for pathway regulation.

Although the role of the PDGFRα signaling/primary cilium in cancer progression remains to be defined, the fact that a loss of the primary cilium can induce either signaling inhibition or hyperactivation indicates that PDGFRα signaling requires the cilium to function correctly.

### 3.3. Wnt Signaling Pathway

Wnt glycoproteins are secreted ligands that are encoded by 19 genes in the human genome [105]. Following the extremely complex process of secretion, Wnts exert their functions by binding to the Frizzled receptors and the lipoprotein-receptor related protein (LRP) co-receptors (LRP 5/6) [106,107,108].

These secreted glycoproteins can differentially activate two distinct signaling pathways that are β-catenin dependent or independent. In the canonical Wnt/β-catenin pathway, binding to Frizzled proteins activates the Dvl protein [109], and this results in the inhibition of the destruction complex containing Adenomatous polyposis coli (Apc), Axin2, Glycogen synthase kinase 3 (Gsk3), and Casein Kinase 1a (CK1a) [110,111,112], that constantly degrades β-catenin in the cytoplasm. Thus, when the pathway is activated, stabilized β-catenin can translocate in the nucleus, where it interacts with other transcription factors belonging to the T-cell factor/lymphoid-enhanced binding factor 1 (TCF/LEF1) family, and it triggers the expression of Wnt target genes involved in cell proliferation, differentiation, and metabolism [113,114]. In the absence of a ligand, Dvl does not inhibit the destruction complex, and consequently, β-catenin is targeted to proteasomal degradation. The non-canonical planar cell polarity (PCP) Wnt pathway ensures cell polarization and orientation in the plane of the epithelium [115]. At the molecular level, the Wnt pathway culminates in cytoskeletal reorganization, and it changes to cell polarity and migration through the activity of Rac and Rho small GTPases [116].

The inversin protein was found to interact with and to inhibit Dvl and Apc2, by acting as a molecular switch between the canonical and non-canonical Wnt pathways [117,118]. Inversin depletion induced PKD in zebrafish and mice by the uncontrolled activation of canonical Wnt signaling, as a possible result of the inhibition of the proteasomal degradation of β-catenin [119]. Since inversin localizes to primary cilia [117], and the aberrant cyst formation due to its inhibition was a phenocopy of the loss of KIF3A-dependent impairment of ciliogenesis [120], it was generally assumed that the primary cilium functioned in hampering the Wnt pathway. In addition, Axin2, Apc, and Vangl2 were found to be localized at the basal body and mutations in the genes encoding basal body proteins impaired both Wnt pathways [29,118,119]. Another study by Corbit et al. favored the restricting function of the primary cilium on the Wnt signaling pathway in mouse embryos, primary fibroblasts, and embryonic stem cells [121]. In these models, a loss of primary cilia induced by mutations affecting Kif3a, Ift88, or oral-facial-digital syndrome 1 (Ofd1) caused Wnt hyper-responsiveness. The loss of Kif3a led to the constitutive phosphorylation of Dvl by CK1, and the subsequent cytoplasmic stabilization of β-catenin was responsible for Wnt pathway hyperactivation. Similarly, it was shown that MEFs treated with *Kif3a* small interfering RNA (siRNA), or with a mutation in the retrograde transport motor dynein cytoplasmic heavy chain 2 (*Dnchc2*) gene showed no ciliation, and an increase in nuclear translocation of β-catenin, by a mechanism involving the Jouberin (Jbn) protein, which is normally restricted to the primary cilium, thus limiting β-catenin nuclear entry [122]. The hyperactivation of Wnt signaling, induced by the loss of the primary cilium, and by the subsequent liberation of Jbn, was also confirmed in colon cancer cells, in which the loss of cilia may represent an important step in the transformation process. Recent work in support of the tumor suppressor activity of primary cilia related to Wnt pathway activation, elegantly describes, both in vitro and in vivo in mice, the histone methyltransferase EZH2 as a driver of melanomagenesis via primary cilia deconstruction [123]. The authors showed that the loss of ciliation-induced Wnt pathway activation, detected by an increase of nuclear non-phosphorylated β-catenin that is responsible for the initiation of metastatic melanoma. Moreover, the loss of primary cilia was found to characterize every stage of human prostate cancer, and unciliated cells were found to have high levels of nuclear β-catenin expression, indicative of Wnt pathway activation [46].

The Wnt pathway is also implicated in the initiation and progression of various types of tumors, most likely through the regulation of β-catenin-responsive genes affecting cell fate, growth, and apoptosis [124]. Although the role of primary cilia in regulating the Wnt pathway is controversial, as demonstrated by some studies in mice and zebrafish in which the impairment of ciliary genes (i.e., *Ift88*, *Ift72*, *Dync2h1* and *Kif3a*) had no effect in Wnt responsiveness or *Axin2* expression [125,126], in cancer, it seems that cilium disassembly acts as a tumor promoter by enhancing Wnt activity and dysregulating cell proliferation and differentiation.

In conclusion, the majority of signaling pathways are directed by the primary cilium, which elicits a cellular response by sensing the extracellular environment. A defective cilium can lead to signaling impairments, causing the induction of tumorigenesis. In cancer, the presence or absence of the primary cilium can trigger or inhibit cancer progression, depending on the type of cancer, the cancer-initiating mutations, and on the altered molecular pathway responsible for tumor insurgence.

## 4. Primary Cilium and Autophagy

Autophagy is a known survival mechanism that is evolutionarily conserved. It involves the formation of autophagosomes for the degradation of macromolecules and organelles via the lysosome, by providing cells with lipids, carbohydrates, amino acids, and nucleotides to maintain cellular homeostasis, and to sustain cellular energetics and development during nutrient scarcity [127,128]. Due to its role in maintaining cellular and genomic integrity, autophagy is thought to prevent cancer development. Conversely, once cancer is established, its function in fueling tumor cells with molecular building blocks as a substrate for biosynthesis and energy, as well as in protecting tumor cells from cell death, makes autophagy a prerequisite for tumor development [129,130]. In this section, we will discuss the connection between primary cilia and autophagy, and how this interplay influences cancer development.

### 4.1. The Primary Cilium Regulates Autophagy

Serum starvation of culture cells is known to both enhance ciliogenesis, by directing cell cycle exit, and autophagy. The first link between primary cilia and autophagy come from the study by Pampliega et al. on MEFs, depleted for Ift20, and kidney epithelial cells (KECs), from *Ift88^−/−^* mice [131]. The resultant impairment of ciliogenesis, due to IFTB component knock out, prevented the full activation of autophagy upon serum starvation, as indicated by the analyses of autophagic flux and autophagolysosome formation. The cilial requirement for autophagy was confirmed by control experiments, in which PDGF-induced cilia resorption decreased starvation-induced autophagy. The mechanism behind this resides in the cilium-dependent Hedgehog pathway: the induction of the Hedgehog pathway in MEFs and KECs cultured in rich medium rescued autophagy to similar levels as those without serum, but only in cells in which ciliogenesis was not compromised. Indeed, in *Ift88^−/−^* cells, only Hedgehog activation by the cilium-independent overexpression of Gli was able to partially rescue autophagy in serum-starved conditions. Several autophagic signaling components were found to be localized to the basal body, and among these, the autophagy-related (Atg) Atg16L protein was recruited only upon serum removal through IFT20-dependent vesicle trafficking from the Golgi to the base of the cilium [132], a novel possible site for autophagosome formation [131]. A positive role for the Hedgehog pathway in autophagy regulation was also found in ciliated neuron and smooth muscle cells [133,134]. The positive role of cilia in autophagy activation was also confirmed by Wang et al., where cilia shortening induced by IFT88 knockdown in human kidney proximal tubular cells impaired autophagy through the activation of mTOR signaling [135]. Although Pampliega et al. did not observe an activation of the mTOR pathway upon cilia-related autophagy inhibition, disruption of ciliogenesis was shown to lead to an increase in the phosphorylation of mTOR and AKT1 [135], the downstream substrates of the mTOR pathway.

### 4.2. Autophagy Regulates Ciliogenesis

**Figure 4:**
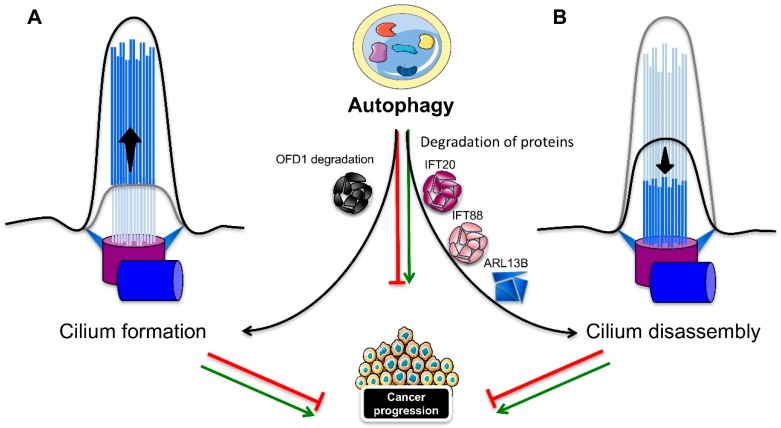
Cilia-autophagy axis. Autophagy has an opposite effect on ciliogenesis: (**A**) Induction of cilia formation, by suppressing negative regulators of ciliogenesis, such as the centriolar satellite OFD1 protein, or (**B**) induction of cilia disassembly, by degrading proteins that are essential for ciliogenesis, such as IFT20, IFT88, and ARL13B. New molecular effectors mediating the cilia-autophagy axis are emerging. Autophagy can both repress and induce cell transformation, but whether and how autophagy influences tumorigenesis through cilia, or vice versa, requires further analysis. ARL13B: ADP ribosylation factor like GTPase 13B; IFT: intraflagellar transport; OFD1: oral-facial-digital syndrome 1.

Autophagy can also positively or negatively influence ciliogenesis and cilia length (Figure 4). Tang et al. showed that the induced autophagy helped to degrade the centriolar satellite pool of OFD1 protein (Figure 4A), which localizes around the basal body [136]. Although OFD1 protein localization at the distal end of the centrioles is required for distal appendage formation during centrosome maturation and for ciliogenesis [136,137], the OFD1 protein at the centriolar satellite seems to inhibit ciliogenesis. Indeed, autophagy compromised *Atg5^−/−^* MEFs with normal levels of Ofd1 showed a marked reduction in primary cilia formation, with a severe decrease in length, but without any impact on cell cycle progression. Moreover, the knockdown of Ofd1 was able to restore ciliogenesis, even in the presence of serum in both the MEFs and MCF7 breast cell lines, thus indicating that OFD1 has a suppressive role on ciliogenesis and that autophagy, by degrading the satellite OFD1, is instead a positive regulator [136]. Conversely, Pampliega et al. found that *Atg5−/−* MEFs were able to more quickly grow primary cilia that were longer and completely functional in serum-rich medium [131]. Autophagy exerted a negative regulation on ciliogenesis through a partial IFT20 degradation (Figure 4B), since *Atg5^−/−^* MEFs or MEFs treated with lysosomal inhibitors showed stabilization of the protein in small cytosolic vesicles. Wang et al. also showed a reciprocal effect between the primary cilium and autophagy: if primary cilia are required for basal and induced autophagy, autophagy is at the same time necessary for ciliogenesis. Autophagy stimulation was able to induce cilia lengthening, whereas the inhibition of autophagy caused cilia shortening in both a murine kidney cell line and tissue [135]. A recent study focused on the mechanism of primary cilia resorption, in concomitance with a gradual decrease of autophagy in human retinal pigmented epithelial (RPE1) cells [138]. A correlation between primary cilia resorption and the inhibition of autophagy in serum-starved cells subjected to gradual serum restimulation was confirmed. Induction of the autophagic flux that followed rapamycin treatment in serum-stimulated cells was partially able to restore proper ciliogenesis. Although the cilia were shorter and fewer compared to the autophagy-induced cilia by serum starvation, rapamycin prevented the disassembly of the primary cilium through the activation of autophagy-dependent ciliogenesis. The same effect of rapamycin on the induction of ciliogenesis and the shortening of cilia was observed by Takahashi et al. [139]. However, the effect of mTOR inactivation induced by glucose deprivation or rapamycin was not the promotion of autophagy, but mainly the upregulation of p27^KIP1^, a cyclin-dependent kinase inhibitor, which thus induced cell cycle arrest. A growing number of studies have characterized the molecular mediators residing in between the autophagy-ciliogenesis axis. The RPGRIP1L ciliary protein was found to positively regulate autophagy through the inhibition of mTOR pathway, and to affect ciliary length [140]. Peroxisome proliferator-activated receptor alpha (PPARA), a hormone transcription factor that is activated during starvation, was shown to be another positive regulator of ciliogenesis, by activating autophagy both in vivo and in vitro; thus, the pharmacological inhibition of autophagy repressed ciliogenesis. The rapamycin-dependent induction of autophagy recovered ciliogenesis in *ppara^−/−^* MEFs. Moreover, contrary to wild-type mice, *ppara^−/−^* mice failed to activate autophagy, in response to starvation, and displayed impaired ciliogenesis, resulting in kidney damage, similar to a ciliopathy. The activation of autophagy rescued the defective phenotypes [141]. Conversely, nuclear receptor subfamily 1, group H, member 4 (NR1H4), another nuclear hormone receptor, but one that is activated in fed conditions, negatively regulates ciliogenesis. In fact, the ligand activation of NR1H4 impaired ciliogenesis in RPE1 and HK2 cells, while the knockdown of NR1H4 restored ciliogenesis and activated autophagy genes, even in media containing serum. In vivo, treatment with an NR1H4 agonist had the same negative effect as *ppara^−/−^* mice on autophagy, ciliogenesis, and kidney function. Thus, ciliogenesis is strictly dependent on nutrient availability through the action of specific transcription factors that regulate autophagy. A role for the extracellular matrix, and in particular, for the major component type-1 collagen (col 1), has been elucidated in the autophagy dependent regulation of ciliogenesis and cilia length. Col 1 was found to be promote cilia growth by repressing HDAC6-mediated autophagy, thus indicating a negative role for autophagy in ciliogenesis [142]. Finally, the GLI2 transcription factor, one of the effectors of Hedgehog signaling, was found to control cell cycle re-entry and cilia length in an autophagy-dependent manner [143]. NIH3T3 fibroblasts lacking Gli2 expression showed longer primary cilia, and increased autophagy, with a reduction in the Ofd1 protein levels in serum-free media, along with a delay in cell cycle re-entry. The lengthening of primary cilia was disrupted by the inhibition of autophagy, while the Kif3a knockdown-dependent ciliary loss rescued the delay in cell cycle re-entry.

### 4.3. The Cilia–Autophagy Axis in Cancer Development

As discussed above, cilia are capable of inducing autophagy that, in turn, has a dual role in cancer development. Studies on autophagy-deficient mice lacking the *Atg5* gene or harboring an *Atg7* liver-specific deletion developed benign liver tumors, whose size was further reduced with the depletion of the ubiquitin-binding protein p62, thus indicating a suppressive role for autophagy in cell transformation [144]. On the other hand, several groups have shown that autophagy can sustain tumor growth in different types of tumors [130,145]. Most cancer cells are characterized by the loss of primary cilia, but they are still capable of high levels of autophagy, even in the absence of cilia, like pancreatic ductal adenocarcinoma (PDAC) tumor cells [48,145]. An explanation may come from a study addressing the fate of cilia in thyroid tumors, which revealed a decrease in the frequency and length of primary cilia in most Hürthle cells found in benign and malignant thyroid diseases, oncocytic variants of papillary carcinoma and primary Hürthle cell tumors. The XTC.UC1 Hürthle cell carcinoma cell line showed high levels of autophagy resulting in the degradation of IFT88 and ARL13B ciliary proteins, thus affecting ciliogenesis (Figure 4B). Consequently, the negative influence of autophagy in ciliogenesis, as well as the increase in autophagy, may be the cause of the poor prognosis characterizing Hürthle cell carcinoma patients [146]. Contrarily, a recent study assessing the function of thioridazine (THIO), an antipsychotic drug, and a potential anti-cancer treatment administered during chemotherapy, through its function in regulating apoptosis [147], indicates a repressive role for the autophagy–cilia crosstalk in tumorigenesis [148]. THIO treatment was able to induce ciliogenesis in lung cancer cells through the promotion of autophagy, by reducing the potential of invasion, proliferation, and the epithelial–mesenchymal transition (EMT) in A549 cancer cells.

Thus, the presence of cilia and activated autophagy may together contribute to the regulation of cancer development, but their outcomes in cancer development are specific to their different tumor contexts. Despite these emerging studies, further analyses are still needed to understand how autophagy influences tumorigenesis through cilia, or vice versa.

## 5. Primary Cilium, Hypoxia, and Cancer Hallmarks

Hypoxia is a common microenvironmental trait of solid tumors, in which highly proliferative cells rapidly grow away from blood vessels, a precluding condition for the diffusion of oxygen in such a poorly vascularized microenvironment [149]. In these disadvantageous circumstances, tumor cells are able to accomplish an adaptive response, resulting in the reprograming of cellular metabolism, the promotion of cell proliferation, resistance to apoptosis, unlimited replication potential, evasion of immune attack, induction of angiogenesis, and ultimately, in the migration towards less hypoxic areas and invasion. All these responses to the hypoxic microenvironment, which are merely the well-known cancer hallmarks [150], are in part orchestrated by hypoxia-inducible factors (HIFs), the master regulators of oxygen homeostasis [149,151].

HIFs are characterized by two subunits, HIFs-α and HIF-β, of which the α subunits (HIF-1α, HIF-2α and HIF-3α) are strictly activated by hypoxia. Changes in cellular oxygen tension (PO_2_) are perceived by two classes of oxygen-sensors, prolyl hydroxylase (PHD) proteins and factor-inhibiting HIF-1 (FIH), which strictly regulate HIF-α activation, depending on the PO_2_ levels. With high PO_2_, PHDs hydroxylate HIFs-α in the oxygen-dependent degradation domain (ODDD), thus enabling rapid interaction with the ubiquitin E3-containing ligase, von Hippel–Lindau (pVHL) [152]. This interaction causes HIFs-α to be ubiquitinated, and thus driven to proteasomal degradation [153,154]. On the other hand, FIH inactivates HIF-1 through the hydroxylation of its carboxy-terminal transcriptional activation domain (C-TAD), preventing its interaction with the co-activator p300 [155]. The gradual decrease in PO_2_, however, causes the inactivation of PHDs and FIH, allowing for the stabilization of HIF-α, which is free to translocate to the nucleus, and to heterodimerize with the constitutively expressed HIF-β. The heterodimer then interacts with cofactors and binds to hypoxia-response elements (HREs) in the regulatory regions of hypoxia-responsive genes, which in turn mediate responses to hypoxia [156,157].

**Figure 5:**
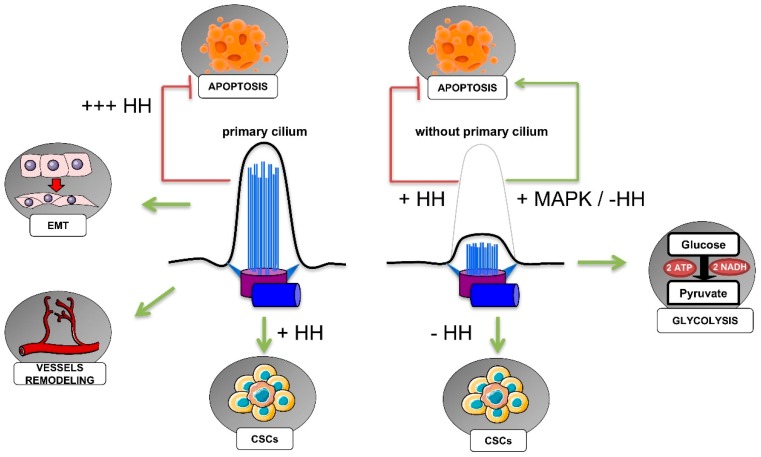
Implication of the primary cilium in cancer hallmarks. In cancer, the presence or absence of the primary cilium can influence cell metabolism, cell invasion, and resistance to cell death, as discussed in the text. Despite the role of the endothelial primary cilia in vessel remodeling, the role of the primary cilium in tumor angiogenesis is not yet known. ATP: Adenosine triphosphate; CSCs: Cancer stem cells; EMT: Epithelial-to-mesenchymal transition; HH: Hedgehog pathway; MAPK: mitogen-activated protein kinase pathway; NADH: Reduced nicotinamide adenine dinucleotide.

In this section, we will describe the role of hypoxia, and of the HIFs in particular, on the regulation of ciliogenesis, as well as what is so far known about the role of the primary cilium in cancer hallmarks (resumed in Figure 5).

### 5.1. HIF-Dependent Regulation of Ciliogenesis in Cancer

Most of our understanding of HIF-dependent regulation of ciliogenesis comes from studies of clear cell renal cell carcinoma (ccRCC), the most frequent histological subtype of renal cell carcinoma (RCC), representing between 70% and 75% of all RCCs [158]. The oncogenic event characterizing both the hereditary and the sporadic form of ccRCC is the biallelic inactivation of the *VHL* gene, which, among its functions, is crucial in addressing HIF-α proteins for proteasomal degradation. ccRCCs are distinguished by cystic precursor lesions that lack cilia [159]. Although the regulation of ciliogenesis by pVHL, a structural component of the cilium [160] is not always considered to be HIF-dependent [161], a negative function was observed for HIF-1α in pVHL-defective ccRCC cells [162]. Overexpression of a mutant pVHL isoform lacking the ability to regulate HIF-α proteins failed to restore cilia, indicating that the loss of cilia in ccRCCs may be a mechanism involving the pVHL inactivation-dependent stabilization of HIF-α. How HIFs mediate cilia resorption is still not completely understood. One explanation could lie in the function of pVHL in regulating the Never in mitosis gene A (NIMA)-related kinase 8 (NEK8) [163]. ccRCC cell lines defective for pVHL showed overexpression of NEK8. Accordingly, *NEK8* expression was found to be increased under hypoxia (1% O_2_), suggesting a role for HIFs in its regulation. This hypothesis was supported by the findings of HIF-1α-specific HREs in *NEK8* promoter. It was further confirmed in vivo in mice exposed to a renal ischemia/reperfusion (I/R) injury. Moreover, the downregulation of NEK8 impaired pVHL knockdown-induced primary cilia resorption, which supports HIF-NEK8 action in the impairment of ciliogenesis. A negative impact of HIF-α on ciliogenesis was also shown in murine bone marrow-derived mesenchymal stem cells (MSC) exposed to hypoxia (1.2% O_2_) [164]. The HIF-1α-dependent disruption of ciliogenesis occurred via Wnt signaling, with HIF-1α inducing the downregulation of the secreted Frizzled-related proteins (sFRP)-1, -3, and -4, and the up-regulation of sFRP-2. Finally, severe cilia disruption was observed in tumors from patients with germline mutations in the pseudohypoxia-linked genes succinate dehydrogenase (*SDHx*) and *VHL* [165], indicating a possible role for the HIFs. Accordingly, pheochromocytoma PC-12 cells showed a decrease in primary cilia expression under hypoxia (1% O_2_), and the same effect was observed with both the downregulation and the pharmacological inhibition of SDHB, and fumarate hydratase (FH). The loss of SDH and FH function induces a pseudohypoxic condition, due to the accumulation of succinate and fumarate oncometabolites that inhibit PHDs and thus pVHL-mediated degradation of HIFα [166]. Thus, HIFα stabilization drove primary cilia loss in pheochromocytoma cells, depending on the activation of the AURKA/HDAC6 cilia resorption pathway [165]. A role for the HIF-induced activation of the AURKA-HDAC6 pathway in ccRCC cells was also demonstrated by Xu et al. [167]. AURKA was regulated by both HIF-1α and HIF-2α subunits, and they also determined the HIF-2α-specific regulation of HEF1, which participates in AURKA activation. The activation of the HEF1-AURKA-HDAC6 pathway had a significant impact on two relevant features of VHL-defective cells: the suppression of primary cilia and the increased of cell motility.

Hypoxia seems to mostly counteract primary cilia formation through the stabilization of HIF transcription factors, following the inactivation of pVHL, which is a structural component of the cilium, and it is essential for ciliogenesis. HIFs are in turn, able to mediate primary cilia disassembly through different pathways, and the role of HIFs in ciliogenesis may be decisive in the adaptation of tumor cells to the hypoxic microenvironment, leading to tumor development.

### 5.2. Primary Cilia in the Regulation of Cancer Cell Metabolism

Cancer cells adapt to hypoxia by shifting their glucose metabolism from aerobic to anaerobic, such as from oxygen-dependent oxidative phosphorylation to oxygen-independent glycolysis, in order to fuel their rapid growth and division [168]. In addition, since the Otto Warburg’s first observations on cancer metabolism, we now know that cancer cells can produce energy for their sustained survival by favoring glycolysis, even in presence of adequate oxygen, leading to a state that has been termed “aerobic glycolysis” [169,170].

There is still little information about the role of primary cilia in the metabolic reprogramming of cancer cells. A recent paper has innovatively investigated the metabolic effects subsequent to the loss of cilia in *IFT88*-deficient thyroid cancer cells. The authors provided evidence that the loss of function (LOF) of *IFT88*/primary cilia results in mitochondrial dysfunction in favor of glycolytic metabolism and lipid biosynthesis [171]. LOF-*IFT88*/primary cilia resulted in mitochondrial fragmentation, impaired oxidative phosphorylation (OXPHOS), diminished mitochondrial membrane potential, and reduced ATP synthesis. On the other hand, increased aerobic glycolysis and fatty acid synthesis was also observed. The results were further confirmed for two out of three thyroid cancer cell lines knocked down for KIF3A, indicating that the shift towards glycolytic metabolism was specifically dependent on the loss of primary cilia.

Besides this above-cited role of the primary cilium in the maintenance of mitochondrial functions, a metabolic shift towards glycolysis has been hypothesized, in correlation with the presence of primary cilia [172]. However, although a cancerous inhibitor of protein phosphatase 2A (CIP2A)-depleted cells showed an increase in primary cilia expression, and in glycolytic activity and capacity, and although the primary cilia seemed to be necessary for enhancing glycolytic activity, the increased glycolytic metabolism was independent to the presence of primary cilia. Nonetheless, this latter study was performed in non-cancerous cell lines, and most of our understanding of primary cilia-dependent metabolic regulation is related to diabetes and obesity [173,174]. Indeed, studies on the implication of primary cilia in the metabolic reprogramming of cancer cells are still limited, and researchers have just started to probe this interesting field.

### 5.3. Primary Cilia, Cancer Stem Cells, and the Epithelial–Mesenchymal Transition

Recent research has revealed a role for hypoxia in the activation and maintenance of cancer stem cells (CSCs) [175,176,177]. CSCs are a subpopulation of cells that are present with varying abundance within most tumors. They are distinguished by the expression of markers that are also expressed in normal stem cells in the tissue of origin. CSCs, thanks to their self-renewal and differentiation capabilities in multiple cell types, have the ability to seed new tumors when implanted in immunodeficient mice, and they give rise to all cell types of a particular tumor sample. CSCs are persistent in the tumor, and given their strong resistance to chemotherapeutic treatments, are the cause of tumor relapse.

Gale et al. have explored CSCs in human and mouse medulloblastomas, as well as the relative presence of primary cilia [178]. Human medulloblastomas CSCs expressing CD133 and CD15 neural stem cell markers were defined by two different populations, with CD15^+^ cells being mostly expressed in recurrent tumors, and characterized by stem-cell properties. As discussed in § 3.1, medulloblastoma insurgence is associated with mutations in the Hedgehog pathway, which is tightly regulated throughout the primary cilium. The authors demonstrated that the Hedgehog pathway and the primary cilium drive the proliferation of human medulloblastoma cells. However, despite the expression of primary cilia in CD15^−^ cells, CD15^+^ cells did not form cilia in the human medulloblastoma, suggesting that this CSC population signaling is independent of the Hedgehog pathway. A recent study has addressed the mechanism inducing CSCs in claudin-low breast cancer [179]. Contrary to that reported by Gate et al. [178], Guen et al. found the Hedgehog pathway and ciliogenesis to be key determinants for driving stemness and tumor growth of the basal compartment [179]. By using HMLE human mammary epithelial cells engineered for the acquisition of an epithelial-to-mesenchymal (EMT) signature, they demonstrated that this HMLE variant was able to strongly increase the expression of primary cilia. The same results were observed for their neoplastic counterparts (HMLER and HMLE cells transformed through H-RAS^G12V^ expression), suggesting that EMT was associated with primary ciliogenesis. Moreover, both cell lines showed activation of the Hedgehog pathway (which was abolished in primary cilia-ablated cells), with GLI2 and GLI3 displaying marked ciliary localization in HMLER cells. Finally, the role of the primary cilium in the acquisition of stemness for normal basal mammary stem cells (MaSCs) was revealed by assessing their capacity to form organoid, and similarly for HMLER CSCs, which showed strong tumor forming capacity after mammary fat pad transplantation. Although it was already known that EMT and the Hedgehog pathway had important roles in the activation of mammary CSCs, this was the first research revealing that the two are interconnected by the primary cilium.

The primary cilium requirement for EMT should not be considered a general rule, and once again, it seems to be related to the type of tissue of cancer onset. In fact, EMT in the kidney is promoted by ARL13B and IFT20 knockdown-induced impairment of ciliogenesis [180], but the study was carried out on the non-cancerous Madin Darby Canine Kidney (MDCK) cell line. Nevertheless, research on the primary cilia–EMT–CSCs axis only at the beginning, and additional studies determining the implication, or not, of the primary cilium will be clinically essential.

### 5.4. Primary Cilia in Cell Death Resistance

One characteristic of tumor cells is their ability to circumvent apoptosis through the imbalance between pro- and anti-apoptotic factors, which enable tumor cells to survive in unfavorable contexts, such as hypoxia and chemotherapy.

Jenks et al. demonstrated an important role for cilia and cilia length in acquired and de novo resistance to different kinase inhibitors, which are clinically relevant therapeutic agents [181]. Several tumor cell lines that become drug-resistant after chronic drug exposure showed a percentage increase in ciliogenesis and/or cilia length, with the appearance of cilia fragmentation. The ciliary length regulator KIF7 was found to be mislocalized at the level of the cilium in the resistant cell lines, and downregulation of the protein in the drug-sensitive cells was sufficient to confer resistance. Changes in cilia length in resistant cells were accompanied by changes in the Hedgehog pathway, resulting in pathway hyperactivation. Finally, targeting ciliogenesis with a small interfering RNA (siRNA) approach or the pharmacological inhibition of the Hedgehog pathway sensitized tumor cells to drugs and enhanced apoptosis. Similarly, cilia shortening following cisplatin treatment in cultured human proximal tubular HK-2 epithelial cells sensitized cells to apoptosis, as indicated by the increase of caspase activity, and genetic ciliary ablation was found to exacerbate this effect [182]. The molecular pathway underlying the cilia-shortening effects on apoptosis was the mitogen-activated protein kinases (MAPK) pathway, and its pharmacological inhibition reverted cilia length and conferred resistance to cell death. The same effect has been seen in a primary gliobastoma cell line depleted for pericentriolar material 1 (PCM1), which showed defects in cilia formation and increased sensitivity to temozolomide by increasing the apoptotic rate [183]. Altogether, these findings on different types of cancer may suggest an innovative approach to resensitizing drug-resistant cancer cells to therapy, by targeting primary cilia and thus triggering cancer cell apoptosis. However, another study has proposed the role of cilia loss in the context of drug resistance to SMO inhibitors in vitro and in vivo, in which the loss of cilia in medulloblastoma cells was associated with mutations in *OFD1*, and a basal level of activated Hedgehog pathway sustained tumor growth [184], indicating that the role of cilia in promoting cell death resistance depends on the oncogenic context.

### 5.5. Primary Cilia in Angiogenesis

The primary cilium has been shown to extend from endothelial cells into the lumen of blood vessels, where it senses the blood flow and when activated, it triggers calcium signaling and nitric oxide production [185]. In the mouse aorta, Dinsmore and Reiter found that primary cilia were enriched at vascular branch points and sites of high curvature, and they assessed the in vivo role of mouse endothelial primary cilia by conditionally depleting *Ift88* [186]. Since primary cilia-enriched areas corresponded to the areas susceptible to atherosclerotic plaque occurrence throughout vasculature, they explored the effects of the absence of endothelial cilia on atherosclerosis. Removal of endothelial cilia in *Apoe^−/−^* mice lacking apolipoprotein E increased atherosclerosis when they were fed with a high-fat, high-cholesterol diet, due to the reduced activity of endothelial nitric oxide synthase (eNOS). In addition to the mechanosensory and anti-atherosclerosis role of endothelial primary cilia, an in vivo ciliary function has recently been found in the formation, remodeling, and maturation of the cranial vascular network in zebrafish [187]. Primary cilia were enriched in nascent vessels independently of their biochemical function as a blood flow sensor, and they were involved in all stages of hindbrain angiogenesis, as suggested by the presence of primary cilia in the region of new central artery sprouting. Mutation or morpholino-induced loss of *ift172* and *ift81* developed cranial hemorrhages in zebrafish embryos, and the phenotype was rescued with the overexpression of *ift81*. The role of endothelial primary cilia in developmental angiogenesis was also confirmed with additional studies in zebrafish and mice, respectively, in the caudal vasculature and in the retina [188,189]. The latter in vivo research on mice demonstrated that the inducible deletion of primary cilia via loss of *Ift88* in retinal endothelial cells triggered premature and random vessel regression, indicating that primary cilia are crucial for proper vascular remodeling [189].

Although the existence of such evidence for primary cilia regulation of developmental angiogenesis, nothing is yet known about the potential function of primary cilia in regulating tumor angiogenesis, such as the formation of new blood vessels in solid tumors in response to hypoxia that enables tumor growth and metastasis. Thus, it will be interesting to address future research in this intriguing field.

## 6. Conclusions

Our knowledge about the primary cilium, a solitary and multitasking organelle, exploded in the second half of last century, and nowadays it is continuing to grow. Besides the sensory role of the primary cilium in olfaction, the perception of light, and mechano- and chemo-perception, it is increasingly being considered to be extremely important for cancer fate.

Cancer outgrowth depends on multiple events, including adaptation to hypoxia, switches in cellular metabolism, escape from apoptosis, autophagy, sustained angiogenesis, and migration, which ultimately lead to metastasis. Both the presence or absence of primary cilia can regulate each event that is essential for cancer survival, thus accentuating our inability to define a precise role for the primary cilium in cancer progression. However, the relationship between the primary cilia and the hypoxic adaptation of cancer cells is an extremely interesting field that requires future investigation.

Primary cilia are interesting targets for novel therapeutic approaches. So-called “ciliotherapy” could be proposed, based on drug repurposing, which would enable the restoration of primary cilia in cilia-lacking cancer cells. On the other hand, primary cilia of ciliated cancer cells may also confer drug resistance to specific kinase inhibitors through the activation of the Hedgehog pathway, and targeting the primary cilium sensitizes cancer cells to cell death. Targeting the primary cilium to turn off the Hedgehog pathway could be another promising therapy. However, the oncogenic mutation-inducing activation of the pathway must be taken into consideration, in order to choose the correct Hedgehog-target drugs to treat individual cancers.

In conclusion, the emerging link between primary cilia and cancer is strictly dependent on tumor types, and it can also vary within the same tumor, or between tumor subtypes. Targeting primary cilia in a therapeutic approach needs to take into consideration all of the events that lead to cancer development, which make the tumor environment extremely heterogeneous (autophagy, hypoxia, EMT, etc.), as well as the degree of ciliation and the tumor-driven oncogenic mutations. Nevertheless, the primary cilium remains a target that should not be neglected, especially in the field of “à la carte” cancer treatment, where the biological profile of the tumors becomes more important than their location, and it is towards this profile that the most innovative treatments are oriented.
